# Institutional delivery and associated factors in rural communities of Central Gondar Zone, Northwest Ethiopia

**DOI:** 10.1371/journal.pone.0255079

**Published:** 2021-07-22

**Authors:** Adane Nigusie, Telake Azale, Mezgebu Yitayal, Lemma Derseh

**Affiliations:** 1 Department of Health Education and Behavioral Science, Institute of Public Health, College of Medicine and Health Sciences, University of Gondar, Gondar, Ethiopia; 2 Department of Health Systems and Policy, Institute of Public Health, College of Medicine and Health Sciences, University of Gondar, Gondar, Ethiopia; 3 Department of Epidemiology and Biostatics, Institute of Public Health, College of Medicine and Health Sciences, University of Gondar, Gondar, Ethiopia; University of Salamanca, SPAIN

## Abstract

**Introduction:**

Institutional delivery has been considered as one of the important strategies to improve maternal and child health and significantly reduces birth-related complications. However, it is still low in developing countries though there are some improvements. Hence, the aim of this study was to assess the prevalence of institutional delivery and associated factors in the study area.

**Methods:**

A community-based cross-sectional study was conducted. A multistage systematic sampling technique was used to select 1,394 study participants. We collected data from 18–48 years old women by using a structured questionnaire. Binary logistic regression was performed to identify factors at 95% confidence level.

**Results:**

The mean age of respondents was 30 (±0.15). The wealth status of 33.48% respondents was poor and 33.33% rich. The prevalence of institutional delivery was 58.17% (95% CI: 55.57%, 60.77%). Multivariable logistic regression showed that demographic factors: women age (≥35years) (AOR = 1.43; 95% CI 1.04, 1.96), having a family size of less than five (AOR = 4.61; 95% CI 3.34, 6.34), having family discussion (AOR = 4.05; 95% CI 2.74, 5.97), distance from the nearby clinic (≤30min) (AOR = 2.92; 95% CI 1.53, 5.58) and decision power about place of delivery (AOR = 2.50; 95% CI 1.56, 4.01); socio-economic factors: husband’s educational status of primary school (AOR = 1.64; 95% CI 1.19, 2.24), middle level household wealth index (AOR = 1.78; 95% CI 1.25, 2.54) and rich level household wealth index (AOR = 2.01; 95% CI 1.42, 2.86); and programmatic factors: antenatal care visit during their recent pregnancy (AOR = 1.86;95% CI 1.16, 2.97) were affects institutional delivery positively. Whereas bad behavior of health workers (AOR = 0.27; 95% CI 0.19, 0.39) negatively affects institutional delivery.

**Conclusion:**

Institutional delivery was low in the study area. This study implies that strengthening family discussion and up taking antenatal care services in regular ways are a few of the suggested recommendations.

## Introduction

Worldwide, to meet the sustainable development goals (SDGs) target, countries with a maternal mortality ratio (MMR) below 432 deaths per 100000 live births in 2015 will need to achieve an annual continuous rate of reduction of 7·5% for 2016–30. Although the rates of reduction that are needed to achieve country-specific SDG targets are ambitious for most high mortality countries, countries that attempted to reduce maternal mortality between 2000 and 2010 provide inspiration and guidance on how to accomplish the acceleration necessary to substantially reduce preventable maternal deaths [1]. Developing regions account for approximately 99% (302 000) of the global maternal deaths in 2015, with sub-Saharan Africa alone accounting for roughly 66% (201 000), followed by Southern Asia (66 000) [2].

Even though several Sub-Saharan African countries achieved a substantial reduction in MMR of nearly 40% since 2000, maternal mortality is higher in women living in rural areas and among poorer communities [1]. For the reduction of maternal mortality, timely use of maternal health services including antenatal care, institutional delivery, and post-natal care was a key strategy. Institutional delivery with the help of skilled health personnel can easily prevent complications of delivery [3–5]. The pregnancy related complications are significantly related to the place of delivery [6]. In connection with this home delivery is associated with much higher risk of neonatal sepsis [7]. According to the 2019 mini-Ethiopian Demographic Health Survey (EDHS) report, the proportion of institutional delivery in the rural areas of Ethiopia was 40%, still lower than other sub-Saharan Africa (SSA) countries which are 61% [8–12]. The primary purpose of institutional delivery was to protect the life of women and their babies and reduce life-threatening conditions through the support and supervision of skilled health care workers [13].

Institutional delivery significantly varied across the residence of women in Ethiopia. As shown by the mini- EDHS 2019 report, and the Central Statistics Agency (CSA), 70% of women who live in urban settings used to deliver in health facilities whereas 40% of women live in rural areas delivered in health facilities [12].

Even though the Ethiopian Federal Ministry of Health has applied different approaches to increase institutional delivery by improving access and strengthening facility-based maternal services, the proportions of institutional delivery is still lower in Ethiopia at the national level [14] than the SSA [9]. Concerning, the Amhara national regional state (ANRS) where this study was conducted, institutional delivery is low (54.2%) as compared to the Health Sector Transformation Plan (HSTP) of Ethiopia [9]. Studies conducted in primary hospital showed that the low quality of prenatal care predisposes women to home delivery [15]. However, this might not be the only reason for low institutional delivery in rural communities.

To back up this, understanding factors that affect institutional delivery is important in order to improve health services delivered to pregnant women, replace home delivery with institutional delivery, and as a result, reduce maternal morbidity and mortality that are related to pregnancy and childbirth.

Even though many studies have been conducted regarding institutional delivery in Ethiopia, the study area is uncovered yet. Therefore, this study was intended to assess the proportion of institutional delivery and identify factors that can significantly predict it, in the context of the Central Gondar Zone.

## Materials and methods

### Study design and setting

A community-based cross-sectional study was conducted among women in 15 rural kebele (the smallest administrative units of Ethiopia) of Central Gondar zone, ANRS, Northwest Ethiopia, from September to December 2019. Central Gondar Zone is found in ANRS and its capital city, Gondar is located 727Kms away from Addis Ababa, the capital city of Ethiopia. According to the 2007 census, the Zone has a population of 2,288,442 inhabitants of whom 462,952 were women of reproductive age. According to the 2019 Central Gondar Zone health department report, there were 14 districts (2 urban and 12 rural), 75 health centers, and 9 hospitals in the zone.

### Study population

All 18–48 years old women who gave birth in the last 12 months before the time of data collection in Central Gondar Zone of selected rural kebeles were included.

### Sample size determinations

We calculated the sample size using single population proportion formula;

n = z (a/2)^2^p (1-p)/d^2^

n = Sample size

Z_a/2_ = 1.96 standard score corresponding to 95% CI

d = 0.03 margin of error

p = 0.271 (Proportion of institutional delivery service utilization in Amhara region) [16].

n = z_(a/2)_^2^p (1-p)/d^2^

n = (1.96)^2^0.271(1-0.271)/(0.03)^2^ = **845**

n = 845, since there is design effect we multiply by 1.5 then it will be 1,267 with 10% non-response rate, it will give 1,394.

Therefore the maximum sample size was 1,394 women who gave birth in the last 12 months.

### Sampling procedure

We employed a multistage sampling technique to select the study participants. By taking 20–30% as a rule of thumb for representativeness, in the first stage among 12 rural districts of the zone we selected randomly two sample rural districts (Dembiya and Wogera) which are composed of 51 kebeles (24 kebele from Dembiya and 27 kebele from Wogera). In the second stage, among the 51 kebeles 15 kebeles were selected with a simple random sampling technique from the two selected districts proportionally. Within the selected kebele, households having the eligible study participants (women who gave birth within the past one year) were identified by using the maternal and child registration book of HEWs. Within the registration book, women who gave birth within the past one year in each kebele were identified before the survey; and we used this as a sampling frame to select the study participants. The HEWs are health cadres who are high school graduates and received one-year training to deliver packages of preventive and health promotion services and few basic curative services.

Then, systematic random sampling was used within k^th^ (5^th^) cases from each kebele to get representative participants until we addressed the required samples. Proportional sampling was done based on the number of women who gave birth in the last year living in the selected kebele using last year’s pregnant women’s registration book as sampling frame in the health post. In connection to this, we selected randomly the first study participant from the list.

### Data collection tool, procedure, and quality control

A structured questionnaire (S1 and S2 Files) was used to collect data which was developed by reviewing the existing body of literature [5, 17–21] and the questionnaire was also piloted (or field-tested) to keep the validity and reliability of the study. The questionnaire was initially prepared in English and translated to the local language Amharic and back-translated to English by language experts to see the consistency. The questionnaire was pretested on 70 women (5% of the entire sample size) in the rural communities of West Gojjam Zone, Bahir Dar Zuria districts which has similar context with the study area.

The data were collected by ten diploma graduate nurses and supervised by three BSc graduate public health officers. Two days training were given for the data collectors and supervisors about the objectives and data collection process by the Principal investigator. The data were checked for accuracy and consistency daily by the field supervisors.

### Study variables

The outcome variable of the study was institutional delivery which was defined as coded as “Yes” if women reported they gave their most recent birth (within the last one year) at a health institution, and “No” if otherwise.

The independent variables of the study includes:- Basic socio-demographic information like: age, ethnicity, religion, marital status, educational status, number of childbirth, occupation of mothers, educational status of women, head of the household, occupation of the husband, family size, and wealth index of households; Health related information and supervision system like: source of information, family discussion, community organization discussion, frequency of supervision by health care providers and community leaders; Predisposing and enabling factors which impede institutional delivery behavior of women like: distance from health facilities, decision power about place of birth, social and cultural influence and, impeding factors; Need related factors like: status of pregnancy i.e. wanted or unwanted pregnancy, ANC follow-up, visiting health facilities during complications, regular check-up of health status, previous place of delivery, and place of delivery for the next pregnancy; Characteristics of health delivery system like: Location of health facility, quality of service, time taken, bad history of health facility, and poor infrastructure; Maternity benefit patterns like: previous place of delivery, place of delivery for the next pregnancy, and reason for home delivery; Courtesy of health service providers like: intention to serve, bad attitude of health workers, and lack of experience.

The wealth index was constructed using a principal component analysis (PCA) after having data on household assets, which included about twenty durable asset lists, recording the land and animals owned, and observing housing materials. Finally, different important family asset factors were summed up to categorize individuals into wealth tertiles (poor, medium, rich).

### Data processing and analysis

The collected data (S3 File) were checked for completeness, consistency, and missing values; coded and entered, using Epi Data version 3.1, and cleaned and analyzed using STATA software version14.1.

Using descriptive methods, the data were summarized, the prevalence of institutional delivery was determined and odds ratios (OR) were obtained using logistic regression. The data obtained from individuals in each household are pooled to create a single large data set then the studies use the number of individuals institutional delivery analyzed as the statistical n value, which is we assume the data gathered at each kebele to be an independent measurement so that we can use simple logistic regression by ignoring clustering [22]. The effect of different variables on institutional delivery was explored using crude and adjusted odds ratios. After checking the correlation of independent variables, significance was determined using crude and adjusted odds ratios with 95% confidence intervals. To determine the association between the different predictor variables with the dependent variable, first bi-variable analysis between each independent variable and outcome variable was investigated using a binary logistic regression model and then all variables having p-value < 0.2 in the bi-variable analysis were suggested as a criterion for variable selection for inclusion into a multivariable model. Hence, all variables with a p-value of < 0.2 in the bi-variable were analyzed for multi-variable logistic regression.

Adjusted Odds Ratios (AOR) with 95% Confidence Intervals (CI) were calculated to show the presence and strength of associations. A variable with a p-value of less than 0.05 in the multivariable logistic regression model was considered statistically significant.

### Ethical approval and consent to participate

Ethical approval was taken from the University of Gondar Institutional review board (protocol number: O/V/P/RCS/05/1048/2019). Official letters were written to the Amhara public health institute. The Amhara public health institute wrote a letter to the respective officials of the Central Gondar zone health department to obtain permission. After giving information and thoroughly explaining the aim of the study to the respective head office, permission was obtained. Written informed consent was taken from participants. Participants were assured that data would not be disclosed to anybody, they were not requested to give their name, and information was kept confidential as well as their privacy.

## Results

### Socio demographic characteristics of the respondents

A total of 1,389 women who gave birth in the last 12 months were interviewed with a 99.6% response rate. The mean age with a Standard deviation (SD) of the respondents was 30 (±0.15). The majority of the respondents 855 (61.56%) were in the age group of 25–34 years. The majority ethnic groups, 1,368 (98.5%) were Amhara. Orthodox Christianity was followed by 1,378 (99.21%). The majority, 1,254 (90.28%) of the study subjects were married.

Regarding educational status, 1,105 (79.55%) of women and 767 (55.22%) of their husbands had no formal education. The majority, 1,254 (90.28%) of the study subjects were housewives in occupation. Eight hundred fifty-seven (61.7%) respondents had a family size of greater than or equal to five. When we see the wealth status of respondent’s households 465 (33.48%) had a wealth status of poor; whereas 461 (33.19%) respondents household had a wealth status of medium and wealth status of rich accounts 463 (33.33%) (Table 1).

**Table 1:** Socio-economic and demographic characteristics of women in Wogera and East Dembiya District, North West Ethiopia, 2020.

Variables		Number	Percent
Age	18–24	170	12.24
	25–34	855	61.56
	35 and above	364	26.21
Religion	Orthodox	1378	99.21
	*^1^Other(Muslim, Catholic)	11	0.79
Ethnicity	Amhara	1368	98.5
	Other(Tigrie, Oromo)	21	1.5
Educational status of the respondent	Unable to read and write	1,105	79.55
	Elementary/Primary	210	15.12
	Secondary school and above	74	5.33
Occupation	Housewife	1,254	90.28
	*^2^Other	135	9.72
Marital status	Currently Married	1,254	90.28
	Currently not Married	135	9.72
Household/family size	1–4	532	38.30
	5 and above	857	61.70
Educational status of the husband(n = 1,254)	Unable to read and write	767	55.22
	Elementary	469	33.77
	Secondary school and above	18	1.30
Wealth status	Poor	465	33.48
	Medium	461	33.19
	Rich	463	33.33

*^1^Other (Muslim, Catholic)

*^2^Other (Petty trade/Student/Laborer)

### Health information and supervision system

This study found that 1,198 (86.25%) of the respondents heard health-related message including institutional delivery within the last three months from the date of the survey, 969 (70%) had a family discussion on the health issue, and 1,129 (81.28%) were frequently supervised by the HEWs and other HWs (Table 2).

**Table 2:** Health information and supervision system in Wogera and East Dembiya District, North west Ethiopia, December 2020.

Variables		Number	Percent
Family discussion on health issue	Yes	969	69.76
	No	420	30.24
Community organization discussion	No org.	181	13.03
	Yes	706	50.83
	No	502	36.14
Frequent supervision by the HDA	Yes	745	53.64
	No	644	46.36
Frequent supervision by the HEW and other HW	Yes	1129	81.28
	No	260	18.72
Heard health related message for the last three month from the date of survey	Yes	1198	86.25
	No	191	13.75

*^1^Other (school adolescent, NGO staff, church/mosque, poster/flyer/leaflet, community event, community discussion, family discussion, clinic/hospital, traditional leader/TBA)

**Fig 1:**
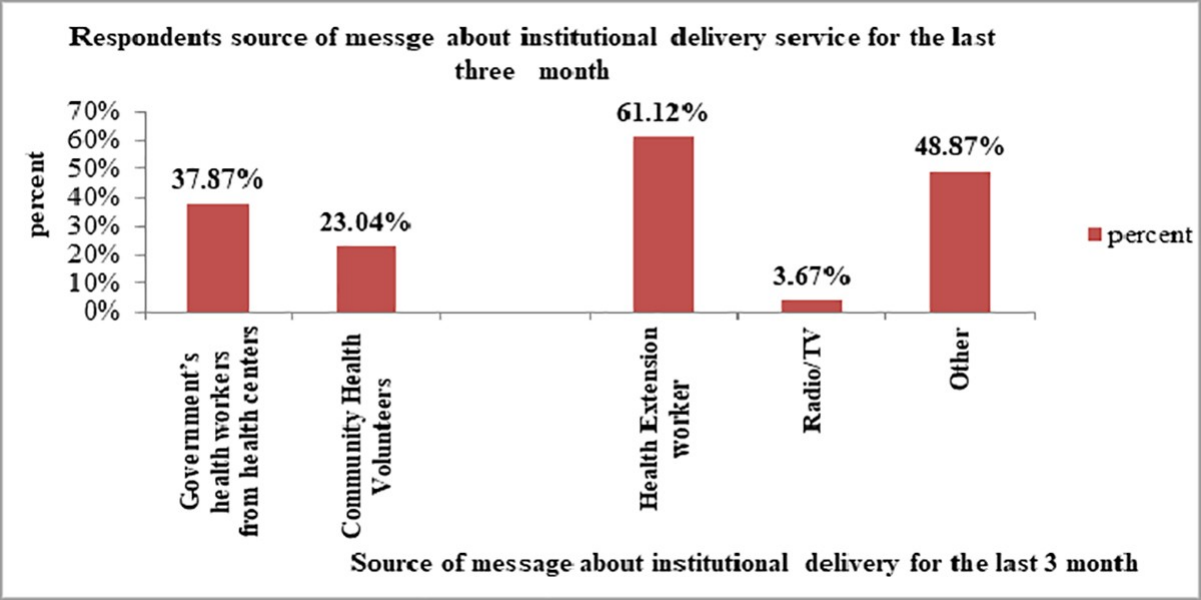
Respondents source of message about institutional delivery for the last 3 month, Central Gondar zone, North West Ethiopia, 2020. Legends *other (school adolescent, NGO staff, church/mosque, poster/flyer/leaflet, community event, community discussion, family discussion, clinic/hospital, traditional leader/TBA).

The study also indicated that HEWs were the main source of message about institutional delivery for the last three month from the date of survey for 61% of the respondents (Fig 1).

### Institutional delivery

The study revealed that 808 (58.17%; 95% CI: 55.57%, 60.77%) of the respondents delivered their last child at health institution with the support of skilled birth attendants, and 1,121 (80.71%) of the respondents intended to give birth for the next pregnancy at the health facility.

The majority, 1,219 (87.76%) of women had at least one ANC visit in which 451 (37%) of them had four or more visits, 536 (44%) two to three visits, followed by 232 (19.03%) one visits. About, 1,299 (93.5%) of the respondents traveled greater than 30 minutes to access the health facility. More than three fourth of the respondents 995 (71.63%) were happy with the services given at the health facility (Table 3).

**Table 3:** Institutional delivery and ANC by women in Wogera and East Dembiya District, North west Ethiopia, 2020.

Variable		Number	Percent
At least one ANC attendant	Yes	1219	87.76
	No	170	12.24
Frequency of ANC (n = 1,219)	1 time	232	19.03
	2–3 time	536	44.0
	4 or more	451	37.0
Place of birth for the last baby	Health facility	808	58.17
	Home	581	41.83
Place of birth for the last baby were the place intended to deliver	Yes	1173	84.45
	No	216	15.55
Intended place for the last baby were (No = 216)	Health facility	166	76.9
	Home	50	23.1
Intended to give birth for the next pregnancy	Health facility	1121	80.71
	Home	268	19.29
Means of transportation for referral case	Own transport	431	31.03
	Public transport	65	4.68
	Ambulance	875	62.99
	*^1^Other	18	1.30
Able to afford transport cost	Yes	842	60.62
	No	547	39.38
Distance of health facility from home	< = 30min	90	6.5
	>30min	1299	93.5
Happy by the services given at health facility	Yes	995	71.63
	No	394	28.37

*^1^other = local ambulance, horse

### Reasons for home delivery

**Fig 2:**
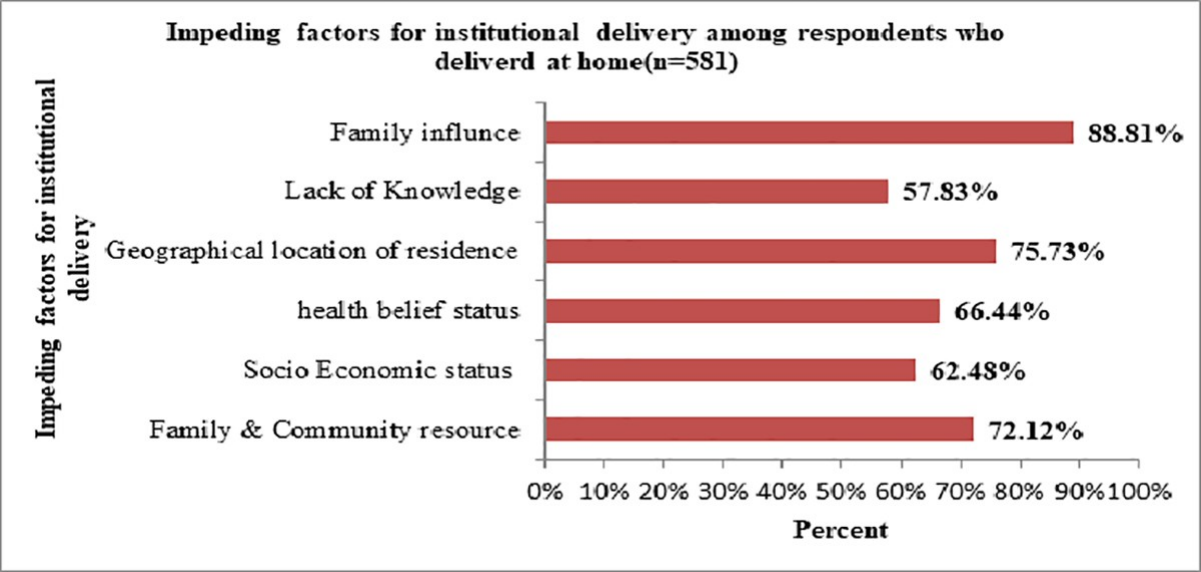
Impeding factors for institutional delivery among respondents who delivered at home, Central Gondar zone, North West Ethiopia, 2020.

Women who did not deliver at health facilities (n = 581) were asked to state their reasons for that. Results in Fig 2 showed that the reason for 516 (88.81%) of respondents for delivering at home was a family influence, two-thirds (66.44%) of these women stated health belief status, (62.48%) socio-economic status, (75.73%) geographical location of residence, (72.12%) family and community resource and (57.83%) lack of knowledge.

Family influence was the most dominant impeding factor for institutional delivery among respondents who delivered at home (Fig 2).

### Factors associated with institutional delivery

In the bivariate analysis, variables such as the age of the respondent, educational status of the respondent, family size, educational status of husband, household wealth index, family discussion, frequently supervised by HEW/HW, ANC Follow up, distance from the nearby clinic (mins), the status of birth, decision power about the place of delivery and bad behavior of health workers were found to have a p-value <0.2 and a candidate for multivariable logistic regression for their significance at p-value < 0.05.

After controlling the effects of potentially confounding variables using multivariable logistic regression model, women age (≥35years), family size (<5), educational status of husband (primary school), household wealth index (middle and rich), family discussion, ANC follow-up, distance from the nearby clinic (<30min), decision power about the place of delivery and bad behavior of health workers were found to be statistically significant predictors of institutional delivery.

The odds of giving birth at a health institution were more than four-and-half folds (AOR = 4.64 [95% CI 3.37, 6.40]) among women who had a family size of less than five as compared to those women who had a family size greater than or equal to five. The odds of institutional delivery were 1.43 times more likely (AOR = 1.43 [95% CI 1.04, 1.96]) among women whose age was > = 35 years compared to women age 25–34 years. Husbands’ educational status was appeared to be associated with deliveries at the health institutions. Women whose husband’s educational status was primary were more than one-and-half fold to give birth at health institutions (AOR = 1.63 [95% CI 1.19, 2.24]) than those women whose husbands educational status was unable to read and write.

Women with a household wealth status of rich and who had a family discussion for their health status in relation to their current pregnancy were two (AOR = 2.01 [95% CI1.42, 2.86]) and four 4.046 (AOR = 4.046 [95% CI: 2.744, 5.967]) folds to give birth at health institution compared to women with a household wealth status of poor and who did not have a family discussion, respectively. Women who attended ANC visit for their current pregnancy, and who spent less than or equal to half an hour to walk to health facility were near to two (AOR = 1.859 [95% CI: 1.164, 2.969]), and almost three (AOR = 2.918; 95% CIL 1.526, 5.581) folds to give birth at health institution compared to women who did not attend ANC visit and who spent greater than half of an hour, respectively.

The odds of giving birth at a health institution were two-and-half folds (AOR = 2.50 [95% CI 1.56, 4.01]) among women who has a decision power about the place of delivery than those who haven’t a decision power about the place of delivery. However, the odds of giving birth at health institution was 73% (AOR = 0.270 [95% CI: 0.186, 0.391]) less likely among women who perceived that the health workers at health facility showed bad behavior and were not available all the time compared to women who perceived that the health workers at health facility did not show bad behavior and were available all the time (Table 4).

**Table 4:** Bivariable and multivariable analysis of factors associated with institutional delivery in Central Gondar zone, Amhara regional state, Northwest Ethiopia 2020.

Variable		Institutional delivery		COR(95%CI)	AOR(95%CI)	P-value
		Yes	No			
Age of the respondent	18–24	95	75	0.97 (0.69, 1.35)	0.65(0.41, 1.01)	0.06
	25–34	484	371	1	1	
	> = 35	229	135	1.30 (1.01, 1.67)*	1.43(1.04, 1.96)*	0.028
Educational status of the respondent	Can’t read and write	600	505	1	1	
	Primary school	152	58	2.21(1.59, 3.05)*	1.48(0.98, 2.23)	0.065
	Secondary school and above	56	18	2.62(1.52, 4.51)*	1.26(0.65, 2.43)	0.498
Family Size	<5	426	106	4.10(3.88, 6.43)*	4.61(3.34, 6.34)*	0.000
	> = 5	382	475	1	1	
Educational status of husband	Can’t read and write	349	418	1	1	
	Primary school	346	123	3.37(2.62, 4.32)*	1.64(1.19, 2.24)*	0.002
	Secondary school and above	12	6	2.40(0.89, 6.45)	1.05(0.32, 3.49)	0.935
House hold Wealth index	Poor	193	272	1	1	
	Middle	331	130	3.59(2.73, 4.72)*	1.78(1.25, 2.54)*	0.001
	Rich	284	179	2.24(1.72, 2.91)*	2.01(1.42, 2.86)*	0.000
Family discussion	Yes	715	254	9.90(7.55, 12.98)*	4.05(2.74, 5.97)*	0.000
	No	93	327	1	1	
Frequently supervised by HEW/HW	Yes	685	444	1.72(1.31, 2.25)*	0.79(0.54, 1.18)	0.252
	No	123	137	1	1	
ANC Follow up	Yes	750	469	3.09(2.20, 4.33)	1.86(1.16, 2.97)*	0.009
	No	58	112	1	1	
Distance from the nearby clinic (mins)	< = 30 min	77	13	4.60(2.53, 8.37)*	2.92(1.53, 5.58)*	0.001
	>30 min	731	568	1	1	
Status of birth	Planned	629	416	1.39(1.09, 1.78)*	1.28(0.92, 1.8)	0.146
	Unplanned	179	165	1	1	
Decision Power about place of delivery	Yes	136	45	2.41(1.69, 3.44)*	2.50(1.56, 4.01)*	0.000
	No	672	536	1	1	
Bad behavior of health workers	Yes	142	273	0.24(0.19, 0.31)*	0.27(0.19, 0.39)*	0.000
	No	666	308	1	1	

* = P-value<0.05

## Discussion

Institutional delivery care service reduces pregnancy related complications, neonatal sepsis and poor birth outcomes [6, 7, 15]. This study has attempted to identify the proportion of women with institutional delivery and associated factors among women who gave birth in the last 12 months prior to the study in 15 rural communities of Central Gondar Zone, Northwest Ethiopia.

The current study showed that the proportion of institutional delivery was 58.17% (95% CI: 55.6%, 60.7%) in the Zone. This finding is consistent with the study conducted in Pawi district (Ethiopia), Kenya, and sub-Saharan Africa where the proportion of women who gave birth on health facilities were 60.5%, 61%, and 57% respectively [8, 13, 23].

Nonetheless, this proportion was better as compared to the national and regional estimates of Ethiopia [12]. The current proportion was also better as compared to a community-based study done in Kersa district, Eastern Ethiopia [24] and a systematic review conducted in Ethiopia [14]. The performance was still below the Health Sector Transformation Plan (HSTP) of Ethiopia in which the plan set to increase institutional deliveries attended by skilled health personnel to 90% [25]. The reason for these differences might be due to the current study was conducted after introducing strong follow-up and supportive supervision strategies on maternal healthcare services in Ethiopia [26]. Conversely, the findings of the current study are lower than studies conducted in other parts of Ethiopia: Mana district, Bench Maji, and Debre Berhan in Ethiopia in which the institutional delivery was 86.4%, 78.3%, and 80.2% respectively [18, 27, 28]. This difference could be due to difference in socio-cultural factors, awareness and knowledge of facility birth, health education, and accessibility of health facility in relation to socio-demographic characteristics [13]. This finding implies that there should be great emphasis on the improvement of accessibility of health facilities integration with the community to increase institutional delivery in the study area.

The current study showed that women whose husbands’ attended primary education were more than one-and-half-fold to give birth at health institutions than those women whose husbands were unable to read and write. This finding was also directly going in line with studies done in other parts of Ethiopia (Mana and Enderta district) and India [28–30]. Husbands with better education may have better access to health-related information that can help them decide their wives deliver at a health facility. Educated husbands are more likely to discuss with their wives on issues, including pregnancy and labour, may give their wives freedom to decide the place of delivery, may insist and support the wives to have a health facility delivery. This indicates that strengthening husband education at the local level via adult education program will help to increase the institutional delivery use since in most of the rural community in the Ethiopia context, husbands were the most influential and major decision-maker of the household activity including health service use.

Women who have ANC follow-up during their recent pregnancy were more likely to deliver in health institutions than those who did not. This result was in line with studies conducted in Pawi district, rural area of Hadya zone, Enderta district, and Bahir Dar in Ethiopia, and Nigeria [13, 19, 20, 31]. ANC can provide an opportunity to promote the benefit of skilled attendance at birth and health facility-based delivery. During ANC visits women are counseled about the benefit of institutional delivery, risk of home delivery, birth preparedness, and complication readiness that can promote women to deliver at a health facility with the help of skilled birth attendants [32–34]. This infers that ANC is an intervention that a pregnant woman receives from organized health care services. The purpose of antenatal care is to prevent or identify and treat conditions that may threaten the health of the fetus/newborn and/or the women and to help a woman approach pregnancy and birth as positive experiences. To a large extent, antenatal care can contribute greatly to this purpose and can in particular help provide a good start for the newborn child. Therefore it is expected to strengthening the antenatal care services for every pregnant women in order to have institutional delivery services for every pregnant.

The odds of institutional delivery were higher among women from rich households compared to women from poor households. The finding is in agreement with the analysis of the 2016 Ethiopian demographic and health survey, and studies conducted in the Mana district of southwest Ethiopia [28], Ghana [35], and Uttarakh [36]. Women from a better wealth status household can cover transportation and other expenses to bring and keep families at a health facility. Besides, women who want to stay at health facilities during the last few days of the pregnancy period for delivery service need to cover the charge of the service they need for the families who may come with them and who stay at home. This finding suggests that the health facility in collaboration with the large community needs to have the establishment of enough waiting home for maternity including for the family members who might be goes with the pregnant women. For such activity, there should be the improvement of community awareness and readiness by providing continuous health education during different devising strategies to improve women’s wealth status, and strengthening resource allocation habit may enhance institutional delivery.

Our study also showed that women’s decision power about the place of delivery was mentioned as influencing factors for institutional delivery of women, which is consistent with previous studies conducted in different areas of Ethiopia [13, 37, 38]. This finding implies that promoting the decision-making power of women for health service use shall be given a great emphasis by the health sector.

Respondents’ age in the current study showed that a positive linear relationship with institutional delivery. This finding is in agreement with a study conducted in the Assosa district [38]. This may be due to women who have a pregnancy at the age of adult were not have a feeling of fear to go to health facilities to give birth [38]. This finding suggests that there should be an awareness creation program for the age group of teenage pregnancy on the use of institutional delivery in a regular way. This means that the accessibility and availability of institutional delivery services including the awareness creation program need to consider the group of individuals who might not have formal marital status within the community.

The influence of traveling time (distance) spent to reach nearby health institutions was the other important factor that was identified by the current study. Women who had to walk thirty minutes or less to reach the health institution were more likely to give birth at health institutions than those who had to travel for greater than thirty minutes. This finding is in line with those of other similar studies done in Ethiopia and other developing countries [28, 39]. It might be related to easily accessible health institutions that may increase the chances of women using it during prenatal periods and at a time of delivery. ANC services and health educations were easily accessible for women whose residence was near to the facility. Furthermore, women who resided in the nearby health facilities had no problem of transportation to attend the institutional delivery service and able to early manage any pregnancy-related problems at any time [13]. This finding may suggest that making health institutions closer to and easily accessible by the communities is very crucial in order to enable more women to give birth at the health institutions.

The current study showed that having a family discussion on health issue including institutional delivery at the household level were more than four folds to give birth at a health facility as compared to their counterparts. This is supported by a finding from Tanzania [40]. This implies that family-level discussion on health issue trajectories including institutional delivery will make them consider health facilities as the best sources of delivery care to ensure safety and pleasant outcomes. This suggests an educational venue that a family could potentially benefit from as far as discussing and deciding about the place of delivery is concerned. Therefore, family-level discussion on the health issue is very important to increasing institutional delivery. So making family-level discussions about delivery and other aspects of health matters should be encouraged within a family level to enhance institutional delivery.

On the other hand, the current study showed that the smaller the family size a woman has, the more the possibility that she would be going to give birth at health facility. This finding is in line with a study conducted in the East Bellesa district [41]. That means, having a family size of smaller is one of the possible reasons for a woman to have freedom in a decision of a place where to give birth which makes a woman feel free about the home activity when they go at a health facility. This may again imply that beyond feeling free of the home activity, then she would tend to be at a health facility in the waiting home for the last week of pregnancy before birth that resulted in a trend association with institutional delivery.

## Strengths and limitations

Since this study is community-based in the rural area, it could reflect the actual experience of women in the community during the study period which could be the strengths of this study.

The potential limitations of this study were that the study is limited to rural women, did not consider the role of husband contribution for institutional delivery, the perception of women on institutional delivery, and the level of community readiness on the promotion of institutional delivery were not addressed.

## Conclusion

Institutional delivery among rural women in the 15 communities of central Gondar Zone had better achievement as compared to the national and regional estimates, but it is still below the HSTP.

Therefore, it should be noted that strengthening ANC follow-up as per the minimum recommended by the WHO will have a positive impact on pregnant women to give birth at health institutions. Promoting husbands’ education at the local level via adult education programs was helpful to encourage women to deliver at health institutions. Moreover, improving and strengthening family level discussion, and empowering women for being a decision-maker in making a choice for a place of delivery is likely to enhance institutional delivery.

## Supporting information

S1 File (PDF)

S2 File (PDF)

S3 File (RAR)

## Abbreviations

ANC: Ante Natal Care
ANRS: Amhara National Regional State
AOR: Adjusted Odds Ratios
BSC: Bachelor of Science
CI: Confidence Intervals
CSA: Central Statistics Agency
EDHS: Ethiopian Demographic Health Survey
HEW: Health Extension Workers
HSTP: Health Sector Transformation Plan
HW: Health Workers
PCA: Principal Component Analysis
SDG: Sustainable Development Goals
SSA: Sub-Saharan Africa
