# Supplementary material for: Institutional delivery and associated factors in rural communities of Central Gondar Zone, Northwest Ethiopia
Source: PLoS One. 2021 Jul 22;16(7):e0255079. doi: 10.1371/journal.pone.0255079 (PMC8297840; doi:10.1371/journal.pone.0255079)
Supplement: S1 File — (PDF) [file pone.0255079.s001.pdf]

Annex II: Participant information sheet and questioners (Amharic)

Participant ID-------------------------------

**የስምምነት ቅጽ**
ጤና ይስጥልኝ እኔ ስሜ------------------ ይባላል የመጣሁት ከጎንደር ዩኒቨርሰቲ ነዉ። የዚህ ጥናትና ምርምር ግብረ ሃይል አባል ስሆን የጥናቱ ዓላማ **በጤና ተቋም የመዉለድ ልምድ በተመለከተ ምን ያህል እንደሆነ እና ተፅዕኖ ሊፈጥሩ የሚችሉ(አስቻይና ገቺ) ምክንያቶችን ለመለየት /ለማወቅ/** መረጃ ለመሰበሰብ እና ለማጥናት የተዘጋጀ ነዉ። እርስዎ እዚህ ጥናት ዉስጥ እንዲካተቱልኝ ሰለመረጥኩዎት ለምጠይቀዎት ጥያቄዎች መረጃዎች በመስጠት እንዲተባበሩኝ በትህትና እጠይቀዎታለሁ። በዚህ መጠይቅ ዉስጥ የሚካተቱ ጥያቄዎች ስለ መሰረታዊ የማህበራዊና ዴሞግራፊያዊ መረጃዎች ፤ የጤና መረጃና ቁጥጥር /ክትትልን በተመለከተ ፤ በጤና ተቋም የወሊድ አገልግሎትን መጠቀም በተመለከተ ፤ እናቶች በጤና ተቋም የወሊድ አገልግሎት የመጠቀም ፍላጎት/መጠቀም መሻት ባህሪ / እንዲኖራቸዉ የሚያነሳሱ/የሚያደርጉ/ አስቻይ እና ገቺ/ፍላጎትን የሚገድቡ/(ተፅዕኖ ሊያደርጉ የሚችሉ)ነገሮችን በተመለከተ ፤ በጤና ተቋም ለመዉለድ ፍላጎትን መሰረት ያደረጉ ጥያቄዎች ፤ የጤና አግልግሎት አሰጣጥን በተመለከተ ፤ የጤና ባለሙያዎቹ ዝንባሌና ፀባይን በተመለከተ እና የመሳሰሉት ተካተውበታል። እርስዎ የሚሰጡት ትክክለኛ መረጃ የጥናትና ምርምራችን ጥራቱን የጠበቀ እንዲሆን ይረዳል። በዚህ መጠይቅ ዉስጥ የእርስዎ መለያዎች የሚሆኑ ማለትም ስምና ትክክለኛ አድራሻ ኣይካተቱም።የሚሰጡን መረጃ ለሌላ ሶስተኛ ወገን ተላልፎ አይሰጥም።<br>የሚሰጡን ትክክለኛ መልሶች ብቻ ናቸዉ የወረዳዉን የእናቶች በጤና ተቋማት የመዉለድ ሁኔታዎችን እንድናዉቅ የሚረዳን። የጥናቱ ተሳታፊ በመሆንዎ በቀጥታ እርሰዎን ተጠቃሚ ሊያደርግ አይችልም ። ነገር ግን የጥናቱ ተሳታፊ በመሆንዎ በአገልግሎት አሰጣጡ ላይ የሚታዩ ችግሮችን በመለየት የተሻለ አገልግሎት እንዲሰጥ ያግዛል ። ስለሆነም ለዚህ ጥናት መሳካት የእርሰዎ አስተዋፅኦ ታላቅ ነዉ።በመሆኑም ለሚየደርጉት አስተዋፅኦዎ በአፀፋዉ ታላቅ ምስጋና አስቀድሜ ማቅረብ እፈልጋለሁ ።<br>የጥናቱን ሀሳብ በትክክል ተረድተዉታል ፤ በጥናቱ ለመሳተፍ ፈቃደኛ ነዎት?<br>1. አዎ   2. አይደለሁም<br>ፈቃደኛ ከሆኑ በፊርማዎ ያረጋግጡልን-------------------------------------

ባለፉት አንድ አመት ጊዜያት ዉስጥ ከወለዱት እናቶች መካከል በጤና ተቋም የመዉለድ ልምድ በተመለከተ ምን ያህል እንደሆነ እና ተፅዕኖ ሊፈጥሩ የሚችሉ(አስቻይና ገቺ) ምክንያቶችን ለመለየት /ለማወቅ/ የተዘጋጀ መጠይቅ፡፡

**የመጠይቁ ኮድ ቁጥር: ....**		**ወረዳ:..........**	**ቀበሌ:.......**	**ቤተሰብ:....**
**ጥያቄ**		**መልስ**		**ዝለል**
**ክፍል I: መሰረታዊ የማህበራዊና ዴሞግራፊያዊ መረጃዎች**				
101.	እድሜ (በአመት)	----------------------		
102.	ብሄር	1. አማራ<br>2. ኦሮሞ<br>3. ትግሬ<br>4. ሌላ, ይገለፅ ______		
103.	ኃይማኖት	1. ኦርቶዶክስ<br>2. ሙስሊም<br>3. ፕሮቴስታንት<br>4. ካቶሊክ 5. ሌላ, ይገለፅ ______		
104.	የትምህርት ደረጃ	1. ማናበብና መፃፍ አልችልም<br>2. ማናበብና መፃፍ እችላለሁ<br>3. 1ኛ ደረጃ (1-8 ክፍል)<br>4. ሁለተኛ ደረጃ (9-10 ክፍል)<br>5. መሰናዶ (11-12 ክፍል)<br>6. ሙያና ቴክኒክ<br>7. ዲፕሎማ ና ከዚያ በላይ		

105.	የጋብቻ ሁኔታ	1. ያላገባ<br>2. ያገባ<br>3. የተፋታ 4. የሞተበት	
106.	ሥራ	1. የቤት እመቤት<br>2. የቀን ሰራተኛ<br>3. ከብት አርቢ/ዘላን<br>4. የግል ሰራተኛ<br>5. የመንግስት ሰራተኛ<br>6. ሌላ ይገለፅ................	
107.	የባል ስራ ምንድነዉ	1. የመንግስት ሰራተኛ<br>2. የግል ተቀጣሪ<br>3. የቀን ሰራተኛ<br>4. ግብርና<br>5. ተማሪ<br>6. ሌላ ይገለፅ................	
108.	ስንት ልጅ ወልደሻል? የሞተ ልጅ አለ	የተወለደ ብዛት------------- የሞተ-----------<br>----	
109.	በበአንድ ቤት ዉስጥ ስንት ሰዉ አብሮ ይኖራል? (በደንብ<br>ይብራራላቸዉ)	----------------------------	
110.	የቤቱ አባወራ ማነዉ?	1. ሚስት<br>2. ባል<br>3. ሌላ፣ ይገለፅ	
111.	የባል የትምህርት ደረጃ	1. ማናበብና መፃፍ አይችልም<br>2. ማናበብና መፃፍ እችላለሁ<br>3. 1ኛ ደረጃ (1-8 ክፍል)<br>4. ሁለተኛ ደረጃ (9-10 ክፍል)<br>5. መሰናዶ (11-12 ክፍል)<br>6. ሙያና ቴክኒክ 7. ዲፕሎማ ና ከዚያ በላይ	

112.	የገቢ መጠን	በሬ		ጤፍ		
		ላም		ዳጉሳ		
		በግ		ስንዴ		
		ፍየል		በቆሎ		
		ፈረስ		ማሽላ		
		በቅሎ		ባቄላ		
		አህያ		አተር		
		ዶሮ		ሽምብራ		
		የቤት		ሬዲዮ		
		የእርሻ መሬት		ስልክ		

ክፍል II : የጤና መረጃና ቁጥጥር /ክትትልን በተመለከተ

201.	ከቤተሰብ አባላት ጋር ስለጤና፤ በጤና ተቋም ስለ መዉለድ፤የቅድመ ወሊድ ክትትል እና በሌሎች ከጤና ጋር በተያያዙ ትወያያለችሁ?	1.አዎ 2. የለም
202.	ከሌሎች የመንግስት አደረጃጀት አባላት ጋር(1ለ5) ስለጤና፤ ተቋም ላይ ስለ መዉለድ፤የቅድመ ወሊድ ክትትል እና በሌሎች ከጤና ጋር በተያያዙ ትወያያለችሁ?	1. አደረጃጀት የለም(1ለ5)<br>2. አዎ<br>3. የለም
203.	የቀበሌ/ማህበረሰብ መሪዎች እና በጎ ፍቃደኞች ተከታታይነት ባለዉ መልኩ ክትትል ያደረጉላችኋል?	1. አዎ 2. የለም

204.	የጤና ኤክስቴንሽን ባለሙያዎች/ሌላ የጤና ባለሙያ በቅርብና ተከታታይነት ባለዉ መልኩ ክትትል ያደረጉላችኋል?	1.አዎ 2.የለም
205.	ላለፉት 3 ወራት ማንኛዉም አይነት መልዕክት ጤናን/ጤና ተቋም ላይ ስለመዉለድ ሰምታችሁ ታዉቃላቸሂሁ?	1.አዎ 2. የለም ***(የለም ከሆነ ወደ ክፍል III)***
206.	ለተ.ቁ.205 መልሱ 'አዎ' ከሆነ, ከየት ነዉ የሰማሽዉ? [ብዙ መልስ መመለስ ይቻላል](ከ 1 በላይ መልስ ይቻላል)	1. ከመንግስት ጤና ባለሙያዎች ከጤና ጣቢያ<br>2. ከማህበረሰብ የጤና በጎፍቃደኞች<br>3. ከጤና ኤክስቴንሽን<br>4. ከተማሪዎች<br>5. መንግስታዊ ካልሆነ ድርጅት ሰራተኞች<br>6. ከቤተክርስቲያን/ከመስጂድ<br>7. ፖስተር/በራሪ ወረቀት/ፍሊፕ ቻርት<br>8. ሬዲዮ/ቴሌቪዥን<br>9. ከማህበረሰብ ዝግጅት/ድግስ<br>10. ከግል/ ከማህበረሰብ ዉይይት /የቡና ዝግጅት<br>11. ከቤተሰብ ዉይይት<br>12. ጤና ጣቢያ/ሆስፒታል<br>13. ከባህላዊ መሪዎች/የልምድ አዋላጅ<br>ሌላ ______________________
207.	ለተ.ቁ.205 መልሱ 'አዎ' ከሆነ, ምን አይነት ጤናን/ጤና ተቋም ላይ መዉለድን/ የተመለከተ መልዕክት እነደሰማህ/ሽ ልታስታዉሽ ትችያለሽ?	______________________________<br>______________________________

ክፍል III : በጤና ተቋም የወሊድ አገልግሎትን መጠቀም በተመለከተ			
301.	ለባለፈዉ የመጨረሻ እርግዝናሽ የቅድመ ወሊድ ክትትል አድርገሽ ታዉቂያለሽ?	1. አዎ<br>2. የለም	(የለም ከሆነ ወደ ጥያቄ ቁ. 303 ሂድ)
302.	ለጥያቄ ቁ.301 መልሱ አዎ ከሆነ፣ለባለፈዉ የመጨረሻ እርግዝናሽ ስንት ጊዜ ተከታትለሻል?	....	
303.	ለጥያቄ ቁ.301 መልሱ የለም ከሆነ፣ለምን?<br>(ከ 1 በላይ መልስ ይቻላል)	1. የቅድመ ወሊድ ክትትል ጥቅም ብዙም አይታየም<br>2. የጤና ተቋሙ ከእኔ ቤት ሩቅ ነዉ.<br>3. ለክፍያ የሚሆን ገንዘብ ስለሌለኝ<br>4. የጤና ባለሙያዎቹ ባህሪይ መጥፎ ነው<br>5. የተዛቡ ባህል ልምዶች/እምነቶች<br>6. ሌላ፣ይገለፅ	
304.	የባለፈዉን የመጨረሻ ልጅሽን የት ነዉ የወለድሽዉ?	1. ከእኔ ቤት<br>2. ከልምድ አዋላጅ ቤት<br>3. ጤና ተቋም<br>4. ሌላ፣ይገለፅ	
305.	ለመዉለድ ያሰብሽበት ቦታ ነዉ የወለድሽዉ (ለጥያቄ ቁ. 304 የመለሽዉ)?	1. አዎ<br>2. የለም	(አዎ ከሆነና የጥያቄ ቁ.304 መልስ ከጤና ተቋም ዉጭ ከሆነ ወደ ጥያቄ ቁ. 307 ሂድ ፤ የጥያቄ ቁ.304 መልስ ጤና ተቋም ከሆነ ወደ ጥያቄ ቁ. 309)
306.	ለጥያቄ ቁ. 305 የለም ከሆነ የት ለመዉለድ ነበር አስበሽ የነበረዉ?	....	

307.	ከጤና ተቋም ዉጭ እንድትወልጂ የዳረገሽ ምክንያት ምንድነዉ (ጥያቄ ቁ.304 ላይ ከጤና ተቋም ዉጭ ላሉ?)	1. ወደ ጤና ተቋም ለመሄድ የትራንስፖርት ችግር<br>2. ጤና ተቋሙ ሩቅ ስለሆነ<br>3. ድንገተኛ ምጥ ስለመጣ<br>4. የጤና ባለሙያዎቹ ባህሪይ መጥፎ መሆን<br>5. ለዘመናዊ ህክምና ዝቅተኛ የሆነ አመኔታ መኖር<br>6. የልምድ አዋላጆች በቤት ዉስጥ እንድንወልድ ስለሚያበረታቱን<br>7. ሌላ፣ይገለፅ	
308.	ለጥያቄ ቁ.304 መልሱ ከጤና ተቋም ዉጭ ከሆነ ማን አዋለደሽ?	1. በልምድ አዋለጅ እርዳታ ከቤቱ<br>2. ያለማንም እርዳታ ከቤቱ<br>3. በባለቤቱ እርዳታ ከቤቱ<br>4. በዘመድ እርዳታ ከቤቱ<br>5. በጤ/ኤክ/ እርዳታ ከቤቱ	
309.	ለቀጣይ የት ነዉ ለመዉለድ ያሰብሽዉ?	1. ጤና ተቋም<br>2. ቤት<br>3. ከባሌ ቤተሰቦች<br>4. ሌላ፣ይገለፅ	
310.	አንድ ነ/ጡር እናት ወደ ወረዳ ሆስፒታል ሪፈር ስትባል በምንድን ትራንስፖርት ነዉ የምትሂደዉ? (ከ 1 በላይ መልስ ይቻላል)	1. በግል ትራንስፖረት<br>2. በህዝብ ትራንስፖረት<br>3. አምቡላንስ<br>4. ሌላ፣ይገለፅ	
311.	ወደ ሌላ ጤና ተቋም ሪፈር በምትባሉበት ጊዜ የትራንስፖርቱን ወጭ መሸፈን ትችላላችሁ?	1. አዎ 2. የለም	አዎ ከሆነ ወደ ጥያቄ ቁ..313 ሂድ
312.	ለጥያቄ ቁ.311 መልሱ 'የለም' ከሆነ ከሚፈለዉ ጤና ተቋም ለመድረስ ምን ታደርጊያለሽ? (ከ 1 በላይ መልስ ይቻላል)	1. ገንዘብ ከጓደኞች/ከጎረቤት እበደራለሁ<br>2. ካለኝ ከብት እሸጣለሁ<br>3. ከመሬቴ ላይ ቆርጨ እሸጣለሁ<br>4. ሪፈሩን አልቀበልም	

313.	የጤና ጠቋሙ ከቤትሽ በአማካይ ምን ያክል ኪ.ሜ ይርቃል? ***(በሚረዱት መልኩ ይብራራላቸዉ)***	1. ኪ.ሜ.............<br>2. ሰዓት	
314.	በጤና ተቋም ላይ በሚሰጠዉ አገልግሎት ደስተኛ ኖዎት? (ብዙ ጊዜ የሚጠቀሙበት ተቋም)	1. አዎ<br>2. አይደለም	አዎ ከሆነ ወደ ጥያቄ ቁ. 316 ሂድ
315.	የጥያቄ ቁ.314 መልሱ 'አይደለም' ከሆነ በሚሰጠዉ አገልግሎት ድስተኛ ያልሆኑበት ምክንያት ምንድነዉ? (ከ 1 በላይ መልስ ይቻላል)	1. የመድሀኒት አቅርቦት አለመኖር<br>2. የባለሙያዎቹ ባህሪይ መጥፎ መሆኑ<br>3. ነፃነት ስለሌለዉ(Lack of privacy)<br>4.የቡና ዝግጅት አለመኖር<br>5. አብረዉ ለሚመጡ ቤተሰቦች በቂ የሆነ ማረፊያ (መኝታ)ስለሌለ<br>6. ሌላ ይገለፅ	
316.	ለምንድነዉ ሁሉም ሌሎች እናቶች በአቅራቢያቸዉ ባለዉ ጤና ተቋም የማይወልዱት ? (ከ 1 በላይ መልስ ይቻላል)	1. ድንገተኛ ምጥ መምጣት<br>2. የባለሙያዎቹ ባህሪይ መጥፎ መሆኑ<br>3. የጤና ተቋሙ ሩቅ መሆን<br>4. የልምድ አዋላጅ መኖር<br>5.የቤተሰብ ተፅዕኖ መኖር<br>6. ሌላ ይገለፅ	
317.	በእናንተ ማህበረሰብ ዉስጥ ከወሊድ በፊት የግድ የሚደረግ ማንኛዉም አይንት ባህላዊ ልምድ አለ ?	....<br>...<br>..<br>.........................	
318.	እናቶች በጤና ተቋም እንዳይወልዱ የሚከለክል በማህበረሰቡ ዉስጥ ያለ ማንኛዉም አይነት ባህላዊ ነገር/ጉዳይ አለ?	....<br>...	
319.	በእርስዎ አስተያየት ጤና ተቋም እየተሰጠ ያለዉን የወሊድ አገልግሎት ይሻሻል ዘንድ ምን ቢሆን ይሻላል ትላህ? (ከ 1 በላይ መልስ ይቻላል)	1. የጤና ባለሙያ ቁጥር መጨመር<br>2. የመድሀኒት አቅርቦትን ማሻሻል<br>3. የጤና ባለሙያዎች እናቶችን ማክበር አለባቸዉ<br>4. አምቡላንስ መኖር አለበት<br>5. ሌላ ይገለፅ	

**ክፍል IV: እናቶች በጤና ተቋም የወሊድ አገልግሎት የመጠቀም ፍላጎት/መጠቀም መሻት ባህሪ / እንዲኖራቸዉ የሚያነሳሱ/የሚያደርጉ/ አስቻይ እና ገቺ/ፍላጎትን የሚገድቡ/ (ተፅዕኖ ሊያደርጉ የሚችሉ)ነገሮችን በተመለከተ**			
401.	የቤተሰብ አባላት ቁጥር	-------------	
402.	የማህበራዊና ባህላዊ እሴቶች በጤና ተቋም ላይ ለመዉለድ ተፅዕኖ ያሳድሩብሻል?	1.አዎ 2.የለም	**(የለም ከሆነ ወደ ጥ.ቁ.404 ሂድ)**
403.	የተ.ቁ.402 መልሱ "አዎ" ከሆነ የማህበራዊና ባህላዊ *እሴቶች* በጤና ተቋም ላይ ለመዉለድ እንዴት ተፅዕኖ ያሳድሩብሻል?	________________________	
404.	ላለፈዉ የመጨረሻ እርግዝናሽ በጤና ተቋም ላለመዉለድ እንቅፋት የሆነብሽ ምንድ ነዉ (ከ 1 በላይ መልስ ይቻላል)?	1. የቤተሰብ ና የማህበረሰብ ሀብት ችግር<br>2. የማህበራዊ ና ኢኮኖሚ ሁኔታ<br>3. ለጤና የሚሰጠዉ እምነት ሁኔታ<br>4. የመኖሪያ ቤቱ አቀማመጥ ሁኔታ<br>5. የእዉቀት ማነስ<br>6. የአመለካከት ችግር	
405.	የት መዉለድ እንዳለብሽ የሚወስነዉ ማነዉ	1. እኔ ራሴ<br>2. ጤና ባለሙያዎች<br>3. ባሌ<br>4. የባሌ ቤተሰቦች<br>5. አያቶቼ<br>6. ሌሎች......	
**ክፍል V: በጤና ተቋም ለመዉለድ ፍላጎትን መሰረት ያደረጉ ጥያቄዎች**			
501.	ሙሉ ጤናማ እያለሽ (ምንም የህመም ስሜት ሳይሰማሽ)ባልተቆራረጠ መንገድ የጤና ሁኔታሽን ትከታተያለሽ	1. አዎ 2. የለም	
502.	በእርግዝናየ ወቅት ወደ ጤና ተቋም የምሄደዉ ችግር/የተባባሰ ህመም ሲሰማኝ ነዉ	1. አዎ 2. የለም	
503.	በማንኛዉም ጊዜ ለሁሉም እርግዝናየ በጤና ተቋም የመዉለድ ፍላጎት/ተነሳሽነት ነዉ ያለኝ	1. አዎ 2. የለም	

504.	የቅድመ ወሊድ ክትትል በተከታታይነት ታደርጊያለሽ?	ı. አዎ 2. የለም	
505.	አሁን ላይ የወለድሽዉ ለመዉለድ ታስቦ ነዉ?	ı. አዎ 2. የለም	
**ክፍል VI: የጤና አግልግሎት አሰጣጥን በተመለከተ**			
601.	የጤና ተቋሙ በጣም ሩቅ ነዉ	ı. አዎ 2. የለም	
602.	በጤና ተቋሙ የሚሠጠዉ አገልግሎት ጥራት በቂ አይደለም	ı. አዎ 2. የለም	
603.	በጤና ተቋሙ የሚሠጠዉ አገልግሎት ዉድና በጣም ጊዜ ወሳጅ ነዉ (የግል/የመንግስት)	ı. አዎ 2. የለም	
604.	ለእኔ ቅርብ የሆነዉ ጤና ተቋም ከአሁን በፊት ጥሩ የሆነ ስም የለዉም(አገልግሎት አሰጣጡ ላይ ችግር አለበት ይባላል)	ı. አዎ 2. የለም	
605.	ስለ ጤና ተቋሙ አገልግሎት የተሟላ መሰረተ ልማትና መረጃ የለም	1. አዎ 2. የለም	
**ክፍል VII: የጤና ባለሙያዎቹ ዝንባሌና ፀባይን በተመለከተ**			
701.	የጤና ባለሙያዎቹ ጥሩ የሆነ አገልግሎት ለመስጠት ተነሳሽነት የላቸዉም	ı. አዎ 2. የለም	
702.	የጤና ባለሙያዎቹ ደንበኛን/ተገልጋይን በማክበር አገልግሎት አየሰጡም / ጥሩ ያልሆነ ስሜት ያሳያሉ	ı. አዎ 2. የለም	
703.	የጤና ባለሙያዎች በማንኛዉም ሰዓት አለመገኘትና መጥፎ የሆነ ባህሪ ማሳየታቸዉ	1. አዎ 2. የለም	

ጥያቄን ጨርሻለሁ፡፡ ሥለ ትብብርዎና ትክክለኛ መረጃዎች በጣም አመሰግናለሁ!!!
