# Supplementary material for: Institutional delivery and associated factors in rural communities of Central Gondar Zone, Northwest Ethiopia
Source: PLoS One. 2021 Jul 22;16(7):e0255079. doi: 10.1371/journal.pone.0255079 (PMC8297840; doi:10.1371/journal.pone.0255079)
Supplement: S2 File — (PDF) [file pone.0255079.s002.pdf]

Annex I: Participant information sheet and questionnaires (English)

Institutional delivery utilization and associated factors in rural communities of Central Gondar zone, northwest Ethiopia

INFORMED CONSENT FORMS
I_____________, the member of the research team would like to inform you that the purpose of this interview is **to determine the prevalence and associated factors of institutional delivry care seeking behavior among mothers who gave birth within the past one year preceding the survey**. Your accurate responses are vital for the quality of our research outputs. Your responses are confidential. By participating in this research there is no potential harm/benefit.<br>Are you willing to participate: Yes______ No_____<br>If you are willing to participate Please put your Signature: _____________.<br>No (Terminate the interview) Interviewer Name __________Signature ________date ______________

INSTRUCTIONS

To determine the prevalence and associated factors of institutional delivery utilization among mothers who gave birth within the past one year preceding the survey; the following questions with different sections are to be addressed to women who gave birth **within the past one year preceding the survey**

Questionnaire Identification Code:		District:	Kebele:	HH:
**Question**		**Response**		**Skip**
**Part I: Basic socio-demographic information**				
101.	What is your age(in years)	----------------------		
102.	Ethnicity	1. Amhara<br>2. Oromo<br>3. Tigray<br>4. others, specify ______		
103.	Religion	1. Orthodox<br>2. Muslim<br>3. Protestant<br>4. Catholic<br>5. Others, specify ______		
104.	Educational status of mother	1. can't read and write<br>2. Can read and write<br>3. primary (1-8 Grade)<br>4. Secondary (9-10 Grade)		

		5. preparatory (11-12 Grade)<br><br>6. technical and vocational schools<br><br>7. tertiary (diploma and above)	
105.	Marital status	1. Single<br><br>2. married<br><br>3. divorced<br><br>4. widowed	
106.	Occupation(Your)	1. House wife<br>2. Peasant<br>3. Pastoralist<br>4. Self employed<br>5. Employed by Government<br>6. Other specify……………	
107.	What is your husband's Occupation	1. Government employee<br>2. Private employee<br>3. Daily laborer<br>4. Farmer<br>5. Student<br>6. Other **(specify)…………………**	
108.	How many children did you give birth?<br><br>Child death?	----------------------------	
109.	How many people are living with you in your house hold? (***Interviewer need to probe***)	----------------------------	
110.	Who is the head of the house hold?	1. Yourself<br>2. Your husband<br>3. Other specify	
111.	Husbands educational level	1. Can't read and write<br><br>2. Can read and write<br><br>3. Grade (1-4th)	

		4. Grade (5-8th)<br>5. Grade (9-12th)<br>6. Grade 12+	
112.	Income /wealth….in number and Kuntal		

Ox		Teff			
Cow		Dagussa			
Sheep		Wheat			
Goat		Bekolo			
Horse		Mashila			
Mule		Bakiela			
Donkey		Ater			
Hen		Shimbira			
No House		Radio			
Farm		Phone			

Part II : Health information and supervision

201.	Have you discussed about health, ID, ANC and other health issues with your family members?	1. Yes<br>2. No	
202.	Have you discussed about health, ID, ANC and other health issues with your 1:5 community organization members?	1. No organization<br>2. Yes<br>3. No	
203.	Are you frequently supervised by the community volunters/ kebele leaders and volunters?	1. Yes 2. No	

204.	Does the health extension worker or other health professional closely supervise you?	1. Yes<br>2. No	
205.	Have you heard any health/ID messages for the last 3 months?	1. Yes<br>2. No	If 'no" go to Part III
206.	If 'Yes' for # 205, from where do you heard? [Multiple answer is possible]	1. Government's health workers from health center<br>2. Community Health Volunteers<br>3. Health Extenstion workers<br>4. School adolescents<br>5. NGO staff<br>6. Church/Mosque<br>7. Poster/flyer/leaflets<br>8. Radio /TV<br>9. Community events<br>10. Private/ community discussion /Coffee cermony<br>11. Family discussion<br>12. Clinic/hospital<br>13. Traditional leader /TBA<br>14. Others ___________________________	
207.	If 'Yes' for # 205, can you tell me what health/ID/ messages you can recall?	___________________________________	

Part III : Institutional delivery service utilization			
301.	Have you ever attended Antenatal clinic in your last pregnancy?	1. Yes<br>2. No (If no skip to question ....303)	
302.	If yes for Q 301, how many times did you attended in the last pregnancy?	..............times	
303.	If no for Q 301why	1. I didn't see any importance of antenatal clinic<br>2. Long distance to health facility from home.<br>3. I had no mony for payement.<br>4. Bad behavior of health workers<br>5.Cultural issue/ belife<br>6. Other specify	
304.	Where did you deliver your last baby?	1. Own home<br>2. TBA's home<br>3. Health facility<br>4. Other specify	
305.	Was that (the answers of Q.304) the place you intended to deliver?	1. Yes<br>2. No	(if **yes** ,and the answer for Q. 304 is other than HF skip to question number 307 but if the answer for Q.304 is at HF skip to Q. 309)
306.	If no for Q305 where did you intended to deliver?	....	
307.	What are the reasons that made you to deliver the place other than Health facility (for those who had delivered out of Health facility?i.e. If the answer for Q.304 is other than HF)	1. Lack of transport to health facility<br>2. Long distance to health facility<br>3. Sudden onset of labour<br>4. Bad behavior of health workers<br>5. Poor belief to modern medicine<br>6. TBA encourage me to give birth at home<br>7. Other specify	

308.	If your delivery is other than HF for Q. 304, who help your birth?	1. In my home with the help of TBA<br>2. In my home without any help<br>3. Home in the help of Husband<br>4. Home in the help of Relatives<br>5. Home in the help of HEW	
309.	Where will you intened to give birth for the next pregnancy?	1. Health facility<br>2. Home<br>3. Husband family<br>4. TBA home<br>5. Other specify	
310.	What is the means of transport when a pregnant mother referred to district hospital?	1. Own transport<br>2. Public transport<br>3. Ambulance<br>4. Other specify ………………..	
311.	Can you able to afford the cost of transport when referred to another health facility?	1. Yes 2. No	If Yes Skip to the next Q.313
312.	If 'No' for Q 312 what will you do to get money to reach a required health facility?	1. Borrowed money from neighbor/ friend<br>2. Sell property<br>3. Refuse referral<br>4. Other specify……………………….	
313.	On average how far is the health facility from your home?<br>(***Interviewr note)***	1. Kilometers………….<br>2. Hours …………….	
314.	Are you happy with the services provided at your health facility?	1. Yes 2. No	If "yes" skip to question 316
315.	If 'no' for Q 314, what things make you unhappy with the services provided at your facilities?	1. No drugs and supplies<br>2. Bad behavior of health workers<br>3. Lack of privacy<br>4. No coffee ceremony<br>5. No place for other member of family where to sleep<br>6. Other specify ……………….	

316.	What makes all other women not deliver in the nearby health facility?	1. Sudden onset of labour<br>2. Bad behavior of health workers<br>3. Long distance to health facility<br>4. Presence of TBA's<br>5.Influnce of other family members<br>6. Other specify	
317.	Is there any traditional habit in your community that should be done before delivery?	....<br>..<br>..<br>..	
318.	Are there any traditional habits that avert women to deliver in health facilities at community?	....<br>..<br>...	
319.	What is your recommendation for improving services in relation to delivery in your health facility?	1. Increase number of health workers<br>2. Improve availability of drugs and supplies<br>3. The health workers should respect the women<br>4. We need ambulance<br>5. Other specify<br>..................................	
**Part IV: Predisposing and Enabling factors which impede ID seeking Behavior of mothers**			
401.	Number of Family member (Family composition)	-------------	
402.	Does the Social & cultural value influence you to give delivery at HF?	1.Yes 2.No	If "no" go to Q.404
403.	If "Yes" for Q.402 How does the social & Cultural value influence you to give birth at HF?	________________________	

404.	Which of the list impede you not to use health facility for delivery service for your last delivery?	1. Family & Community resource<br>2. Socio Economic status<br>3. health belief status<br>4. Geographical location of residence<br>5. Lack of Knowledge<br>6. Attitudinal problem	
405.	Who was decision maker about the place of delivery for the last birth?	1. My self<br>2. Health workers<br>3. My husband<br>4. My husband family<br>5. Mother-in-law<br>6. Others_________	
**Part V: Need related factors questions to bring ID**			
501.	Have you going to check your health status in regular way while you are not face any unhealthy behavior	1. Yes 2. No	
502.	I need to go in health facility when I am facing complication/Problem during pregnancy	1. Yes 2.No	
503.	Whatever the case I have an Intention to deliver at HF for every pregnancy	1. Yes 2.No	
504.	Have you going for regular ANC visit?	1. Yes 2. No	
505.	Does your Pregnancy is Planned or unplanned?	1. Yes 2.No	
**Part VI: Characteristics of health delivery system**			
601.	The Location of health facility is too far	1. Yes 2. No	
602.	The Quality of service given in health facility is not good	1. Yes 2. No	
603.	The service given in the health facility takes **too much time** and costly (public or private)	1. Yes<br>2. 2.No	

604.	The health facility near to me has not a good history in delivery Practice pattern(has bad history)	1. Yes<br>2. No	
605.	Poor Infrastructure and information delivery system in the facility	1. Yes<br>2. No	
**Part VII: Attitude & courtesy of health service provider**			
701.	The health worker has no Initiation to serve	1. Yes<br>2. No	
702.	The health workers had not respectfully deliver the health care services<br>(Bad attitude of Health workers)	1. Yes<br>2. No	
703.	The health workers were not present at any time to give the service fully and show bad behaviour.	1. Yes<br>2. No	

I have finished my interview, thank you for your cooperation!!!
